# Two-Tier Anomaly Detection for V2I Alerting on IoT Vehicle Counts: A Kuwait Corridor Benchmark

**DOI:** 10.3390/s26175368

**Published:** 2026-08-25

**Authors:** Yousef AlSaqabi

**Affiliations:** Department of Electrical Engineering, Kuwait University, Kuwait City 72304, Kuwait; yousef.alsaqabi@ku.edu.kw

**Keywords:** traffic anomaly detection, Internet of Things, inductive loop sensors, CUSUM, Isolation Forest, LSTM autoencoder, vehicle-to-infrastructure (V2I), intelligent transportation systems, smart mobility, Gulf region

## Abstract

**Highlights:**

**What are the main findings?**

- Across ten injection seeds, a training-free CUSUM control chart was the most accurate detector of injected traffic-count anomalies (mean F1 = 0.945, perfect precision on every seed), ahead of Isolation Forest and an LSTM autoencoder.
- In simulation calibrated to the observed traffic, a sub-second edge detector met the 13.5 s vehicle-to-infrastructure (V2I) alerting budget for traffic surges (median 6.8 s at peak rates, robust to signal-cycle platooning), while flow-cutoff detection required roughly 21 s.

**What are the implications of the main findings?**

- Lightweight, training-free detectors are a practical, competitive alternative to datahungry deep-learning models for low-resource roadside hardware.
- For timely V2I alerts, the limiting factor is how quickly an anomaly can be detected, not how quickly the alert can be transmitted.

**Abstract:**

Anomaly detection in vehicular networks focuses on cybersecurity, leaving physical traffic-flow anomalies at urban intersections underserved by IoT sensing. This paper benchmarks anomaly detection on five days of hourly vehicle counts from four consecutive signalized intersections in Kuwait City, with a vehicle-to-infrastructure (V2I) latency feasibility analysis. Anomalies are synthetically injected because verified incident labels are unavailable; scores reflect detectability under the injection protocol rather than validated incident detection. Across ten injection seeds, CUSUM is the most accurate (mean F1 0.945, perfect precision on every seed), Isolation Forest attains the highest recall (0.955), and the LSTM-AE reaches F1 0.347; on misaligned anomaly classes, the margin narrows, and the LSTM-AE matches CUSUM on gradual drift. A corridor rule localizes detected corridor anomalies (9/9, conditional on detection). Hourly aggregation alone imposes an expected 1800 s detection delay, over 130 times the 13.5 s V2I budget at 80 km/h. A sub-second Tier-1 edge detector, evaluated in traffic-calibrated simulation, detects surges within budget (median 6.8 to 9.1 s, robust to signal-cycle platooning), whereas flow-cutoff detection requires roughly 21 s and overnight hours remain a blind spot. Results support a two-tier edge-cloud design and provide, to our knowledge, the first such benchmark on real Gulf-region corridor count data.

## 1. Introduction

Urban road networks in high-density cities are increasingly vulnerable to physical traffic anomalies, including sudden flow disruptions, inter-intersection blockages, and incident-induced surges, which propagate rapidly and challenge real-time safety response [1,2]. Countries in the Middle East face this challenge acutely, driven by extreme motorization rates and limited public transit alternatives. Kuwait exemplifies this condition: with 560 registered vehicles per 1000 residents, roughly triple the global average [3,4], private cars account for approximately 85% of commuting trips in Kuwait City [5], and road traffic fatalities reached an estimated 9.2 per 100,000 population in 2021 [6]. Such disruptions are difficult to detect and respond to in real time, compounding both congestion and safety risk. Automated, infrastructure-level anomaly detection is therefore an operational priority for high-density Gulf urban corridors.

The deployment of IoT-enabled inductive loop sensors at signalized intersections provides a continuous stream of directional vehicle-count data at hourly or sub-hourly granularity [7]. Unlike camera- or GPS-based systems, loop sensor networks operate independently of lighting conditions, weather, and privacy constraints, making them suitable for permanent infrastructure-level deployment [8]. Despite widespread installation in urban ITS infrastructure, the count data produced by these sensors remain largely underutilized for real-time anomaly detection, particularly in the context of multi-intersection corridor monitoring and vehicle-to-infrastructure (V2I) safety integration. Kuwait Vision 2035 identifies smart mobility and intelligent infrastructure as national development priorities [9], yet IoT-enabled anomaly detection integrated with V2I alerting remains absent from the region’s ITS research landscape.

Anomaly detection in vehicular networks is dominated by cybersecurity framing, targeting spoofed messages, Sybil attacks, and adversarial inputs to cooperative perception systems [7,10]. Physical traffic-flow anomalies, such as incidents, surges, and inter-intersection blockages, have received comparatively limited attention from an IoT sensor perspective. Existing physical detection studies predominantly operate on single intersections [8,11], rely on camera- or GPS-based sensing modalities [11], or are evaluated on freeway and arterial highway datasets rather than signalized urban corridor environments [2,12].

V2I communication via Dedicated Short-Range Communication (DSRC) or Cellular V2X (C-V2X) in the 5.9 GHz ITS band enables roadside units (RSUs) to broadcast safety alerts to approaching vehicles with millisecond-level transmission latency [13,14]. However, the end-to-end alerting delay is dominated not by the communication link but by the detection latency, that is, the time required to identify an anomaly from sensor data. At urban arterial speeds, a vehicle traverses a typical RSU broadcast range in approximately 13.5 s [15]. This imposes a hard real-time constraint on the entire detection-to-broadcast pipeline that hourly IoT frameworks do not natively satisfy, motivating a layered edge–cloud detection architecture.

No prior work has combined multi-intersection IoT loop-sensor data, corridor-aware spatial analysis, V2I latency feasibility analysis, and Gulf-region real vehicle-count data within a single study. The five-day window is not presented as sufficient for generalization; it is sufficient for this paper’s stated purpose, a proof-of-concept benchmark of the two-tier framework, because it contains one complete Gulf weekday–weekend cycle, both traffic regimes required by the frozen-baseline protocol, and enough clean hours to calibrate every detector under identical conditions. This paper addresses the identified gap through four contributions:

- *C1—Multi-method benchmark:* A comparative evaluation of CUSUM, Isolation Forest, and LSTM-AE on real IoT loop-sensor vehicle counts from a high-density Kuwaiti urban corridor, using synthetically injected anomalies evaluated across ten independent injection seeds, providing, to our knowledge, the first such benchmark on Gulf-region count data.
- *C2—Corridor-aware localization:* A two-stage upstream-consistency rule that localizes *detected* corridor anomalies to specific inter-intersection segments, demonstrated as a proof of concept on a small, detection-conditioned sample (nine of ten injected corridor anomalies). An ablation shows that raw corridor-ratio features degrade detection, so the mechanism operates as a post-detection localization step rather than as a spatial-fusion detector.
- *C3—V2I latency feasibility analysis:* A quantitative analysis of detection-to-broadcast latency for each method against the 13.5 s V2I alerting requirement at 80 km/h, from which a heuristic Tier-1 edge observation budget of approximately 11.8 s (nine vehicles) is derived as a design target. A sub-second Tier-1 CUSUM detector is then implemented and evaluated in simulation calibrated to the observed arrival rates, quantifying which anomaly classes meet the alerting budget and which remain detection-limited.
- *C4—Gulf-region case study:* To our knowledge, this is the first comparative physical traffic anomaly-detection benchmark on real vehicle-count data from a high-motorization Gulf city, addressing a documented gap in geographically diverse ITS benchmarking [16,17].

The remainder of this paper is organized as follows. Section 2 reviews related work. Section 3 presents the system model. Section 4 describes the detection methodology. Section 5 reports results and discussion. Section 6 analyzes V2I feasibility. Section 7 states the limitations and threats to validity. Section 8 concludes the paper.

## 2. Related Work

### 2.1. Physical Traffic Anomaly Detection

Physical traffic anomaly detection seeks to identify unusual deviations in traffic-flow patterns, such as sudden volume drops, unexpected surges, or atypical temporal profiles, using data from roadway sensors. This domain is distinct from the cybersecurity-oriented anomaly detection that dominates the vehicle-to-everything (V2X) literature, where the focus falls on detecting spoofed or malicious messages rather than physical flow irregularities. Cook et al. [7] provide a comprehensive survey of anomaly detection techniques for IoT time-series data, establishing taxonomies of point, contextual, and collective anomalies that apply directly to traffic count streams. Within the urban traffic domain specifically, Mao et al. [1] address anomaly detection using traffic-monitoring data in urban settings, representing one of relatively few studies that move beyond highway-centric scenarios. Earlier work by Rizvi et al. [8] demonstrates threshold-based incident detection using loop detector data, providing a baseline for real-time capacity estimation but relying on comparatively simple decision rules.

More recent studies leverage IoT sensing and machine learning to improve detection accuracy. Balasubramanian et al. [18] develop an IoT-based system combining machine learning with driver alert functionality, making it one of few works that integrate anomaly detection with downstream notification, an architecture conceptually aligned with V2I alerting. He et al. [2] propose an unsupervised reinforcement learning approach for autonomous anomaly detection on traffic-flow time series, incorporating synthetic anomaly injection on count data to evaluate detection performance. Nouri et al. [19] extend the scope to network-level temporal anomaly detection during extreme events, operating at a broader spatial scale than single-intersection studies. Despite these advances, most existing systems evaluate detection at a single intersection or isolated sensor location, and few quantify the communication latency required to translate a detection event into an actionable driver alert.

The closest prior work to the present study is the Passable system proposed by Alzamzami et al. [11], which integrates incident detection with vehicle alerting at a signalized intersection. Passable employs camera-based sensing and operates within a single-intersection scope, providing an end-to-end pipeline from detection to alert. However, it does not address multi-intersection corridor analysis, does not employ IoT inductive loop-sensor data, and does not include a V2I communication latency feasibility assessment. The present work extends this line of research by introducing corridor-level spatial reasoning across consecutive intersections and by quantifying the latency gap between detection and the V2I alerting requirement.

Existing traffic anomaly detection literature predominantly draws on Western datasets, such as METR-LA, PeMS, and NGSIM, or on Asian urban networks. Traffic studies in Gulf Cooperation Council (GCC) countries remain comparatively sparse and focus on adjacent problems rather than anomaly detection with V2I integration. Almomany et al. [20] apply reinforcement learning to traffic signal optimization in Kuwait City, underscoring the density pressures motivating IoT-based monitoring. AlSaqabi [21] extends RL-based control to IoT-enabled intersections in the same city, yet physical anomaly detection with V2I integration remains unaddressed in the region. Related GCC studies address adjacent problems, including congestion analysis in Saudi Arabia [22], ITS deployment challenges in Qatar [16], and driving-behavior safety analysis in Kuwait [17], none of which include a V2I component. Collectively, these studies confirm that Gulf-region traffic research has not yet engaged physical anomaly detection with V2I alert integration on multi-intersection IoT sensor data, a gap the present study addresses.

### 2.2. Multi-Intersection Spatial Correlation and Detection Methods

Adjacent intersections in urban road networks exhibit strong spatiotemporal flow correlations that decay with topological distance. Ermagun et al. [23] report average local correlations of 0.83 within first-order neighborhoods, establishing that upstream and downstream links carry predictive information about one another. Recent anomaly detection frameworks exploit this structure directly. Zhang et al. [24] employ graph attention mechanisms to capture how anomalous conditions propagate to neighboring nodes in a road network, while Zhao et al. [25] integrate geographic knowledge with tensor decomposition to show that anomalies propagate upstream and downstream with topology-dependent intensity. Liu et al. [26] further demonstrate through a multi-graph formulation that inter-intersection correlations are multi-dimensional, encompassing both static connectivity and dynamic flow coupling. The exploitation of such spatial structure for corridor-aware anomaly detection remains underexplored in the IoT sensor literature, where most studies treat each intersection independently.

The choice of detection algorithms in this study is guided by the principle that no single method dominates across all datasets and anomaly types. Schmidl et al. [27] benchmark 71 algorithms on diverse time series and confirm this finding, which directly motivates a multi-method comparative design. More expressive spatiotemporal models, including Transformer-based detectors, temporal convolutional networks (TCNs), graph attention networks, spatio-temporal graph convolutional networks (STGCN), and diffusion convolutional recurrent networks (DCRNN), achieve strong accuracy on large traffic benchmarks such as METR-LA and PeMS, but such models are typically trained on weeks to months of data across hundreds of sensors. With 480 intersection-hour slots and 460 training sequences available here, any such model would operate at a small fraction of its intended data regime, and a benchmark entry trained far outside that regime would mischaracterize the method class rather than represent it; the LSTM-AE is retained as the deep-learning representative precisely to document this data-regime effect. The present study therefore focuses on lightweight detectors that are deployable on RSU edge hardware, quantifies their computational footprint in Section 5.7, and positions the heavier spatiotemporal models as comparison baselines for future, data-rich extensions. Three complementary paradigms are selected. First, the Cumulative Sum (CUSUM) control chart provides a parameter-light statistical baseline that requires no training data and is well suited to streaming sensor inputs with low computational overhead. Second, the Isolation Forest (IF) [28] offers an unsupervised, multivariate detector with linear-time complexity; its Extended variant [29] resolves the axis-aligned splitting artifacts of the original algorithm. Belay et al. [30] compare 13 unsupervised methods on IoT benchmark datasets (SWaT, SMD) and find IF competitive with more complex deep-learning alternatives, supporting its selection for resource-constrained IoT deployments. Third, the LSTM autoencoder (LSTM-AE) captures temporal dependencies in sequential data and flags anomalies via reconstruction-error thresholding [10,31]. Together, these three methods span statistical, tree-based, and deep-learning paradigms, enabling a principled benchmark comparison.

Evaluating unsupervised anomaly detectors on real traffic data poses a fundamental challenge: ground-truth anomaly labels are rarely available. Synthetic anomaly injection into clean real-world data is therefore the standard evaluation protocol [32]. However, the widely used point-adjust F1 metric inflates scores by granting full credit when any point in an anomalous segment is detected. Liu and Paparrizos [33] expose this bias and recommend range-aware alternatives such as VUS-PR. The experimental design in this study draws on corrected injection methodology [32,33] to support benchmark reliability.

### 2.3. V2I Communication Standards and Latency

Vehicle-to-infrastructure (V2I) communication operates primarily through two competing technologies in the 5.9 GHz ITS band: Dedicated Short-Range Communications (DSRC), standardized as IEEE 802.11p, and Cellular V2X (C-V2X) [13]. Both technologies have reached deployment maturity, with field trials confirming their viability in urban environments [34]. Empirical performance comparisons demonstrate that DSRC achieves shorter end-to-end latency than LTE-V2X in urban V2I scenarios [14], although C-V2X offers advantages in coverage and network management. Field measurements of IEEE 802.11p roadside units (RSUs) report effective broadcast ranges of 300–500 m under line-of-sight conditions [35], a parameter that informs the RSU spacing assumptions adopted in the feasibility analysis of Section 6. Both technologies operate in the 5.9 GHz ITS band and satisfy the latency requirements relevant to this work [13].

The authoritative latency budget for V2I safety applications is defined by 3GPP TS 22.185, which specifies an end-to-end requirement of ≤100 ms for general V2X safety services and ≤20 ms for pre-crash scenarios [15]. 3GPP TR 22.886 introduces tiered requirements ranging from 25 ms for lower levels of automation to 6 ms for higher levels [36]. Critically, multi-access edge computing (MEC) deployment reduces network-side latency to approximately 8 ms compared with roughly 54 ms for cloud-based processing [37], motivating the edge-tier architecture proposed in this study. Resource allocation across LTE-V2X and NR-V2X modes must satisfy these latency constraints, as surveyed by Khabaz et al. [38].

However, the communication link itself is not the latency bottleneck. Detection latency, the time from anomaly onset to alert generation, dominates the end-to-end delay budget. Even in dedicated V2I incident detection systems, detection latency of 20–30 s has been reported for high-accuracy methods [12], which far exceeds the real-time budget on high-speed arterials. This disparity between communication capability and detection speed motivates the V2I feasibility analysis and two-tier architecture developed in Section 6.

### 2.4. Positioning of the Present Work

Table 1 summarizes the key dimensions along which the present study is differentiated from representative prior work. Existing physical traffic anomaly detection studies address either single-intersection detection without V2I integration [2,8,11], or network-level detection without corridor-aware spatial reasoning or latency analysis [1,19]. The closest prior work, Passable [11], integrates detection with vehicle alerting but operates on camera data at a single intersection and provides no V2I latency feasibility assessment. No prior study combines multi-intersection IoT inductive loop-sensor data, corridor-aware spatial reasoning, V2I latency analysis, and real-world Gulf-region data within a single study. The present work addresses this intersection of gaps through Contributions C1–C4.

## 3. System Model

This section describes the physical sensor deployment, the collected dataset, and the end-to-end V2I alert pipeline that together constitute the proposed system.

### 3.1. Physical Deployment

The study corridor comprises four consecutive signalized intersections, designated I1 through I4, situated along the Second Ring Road, a high-density urban arterial in Kuwait City. Intersections I1, I2, and I3 are standard four-way junctions, whereas I4 is a three-way junction. Each intersection is equipped with in-road inductive loop sensors recording directional vehicle counts from four approach arms at I1–I3 and three at I4. Sensor observations are forwarded to an edge processing module co-located with a roadside unit (RSU) at each site, forming the first layer of a tiered anomaly detection and V2I alerting architecture.

### 3.2. Dataset Description

Data were collected over five consecutive days from 15 August to 19 August 2025. Under the Gulf calendar, this period spans two weekend days (Friday 15 August and Saturday 16 August) and three weekdays (Sunday 17 August through Tuesday 19 August). All sensors recorded hourly vehicle counts over each 24 h cycle, yielding 15 directional streams: four streams at each of I1, I2, and I3, and three streams at I4, for a total of 1800 observations. The dataset contained zero missing values and zero sensor dropouts across all streams. The same dataset was also used to evaluate a reinforcement learning-based adaptive signal controller at this corridor [21].

Daily aggregate volumes revealed a pronounced weekday–weekend contrast. Intersections I1 through I3 exhibited a weekday volume increase of 32–51% relative to weekend days, while I4 showed a substantially larger lift of 109%, attributable to its proximity to weekday-dominant traffic generators. Peak throughput occurred at 20:00 for I1–I3 and at 18:00 for I4, with the highest single-hour count of 2102 vehicles recorded on the southbound approach of I2. The late-evening peak reflects the characteristic Gulf urban activity pattern, in which commercial and social travel is concentrated after sunset during summer months [20]. This unimodal evening profile contrasts with the bimodal morning–evening commute pattern characteristic of Western urban traffic datasets such as METR-LA and PeMS, underscoring the value of region-specific data for traffic anomaly detection research.

### 3.3. V2I Alert Pipeline

The proposed system operates as a three-tier pipeline. At the *sensing tier*, the IoT vehicle-count sensors at each intersection transmit raw directional counts to the co-located edge processor at a fixed hourly interval. At the *detection tier*, the edge processor executes the corridor-aware anomaly detection framework detailed in Section 4, evaluating incoming counts against learned normal profiles and upstream–downstream spatial consistency checks. When the detection module confirms an anomaly, the *alerting tier* is activated: the RSU broadcasts a V2I safety alert to approaching vehicles via DSRC or C-V2X operating in the 5.9 GHz ITS band [13,14]. Because the detection framework operates on hourly aggregates, the end-to-end latency of this tier exceeds real-time requirements for safety-critical applications. Beyond direct driver warnings, broadcast anomaly alerts can also serve as inputs to vehicle-side decision layers, such as data-aware path planning for autonomous vehicles [39]. A complementary streaming Tier-1 detector operating at sub-second granularity is therefore necessary; the feasibility of meeting the requisite latency budget is analyzed and the Tier-1 detector implemented and evaluated in simulation in Section 6.

### 3.4. Corridor Architecture

**Figure 1:**
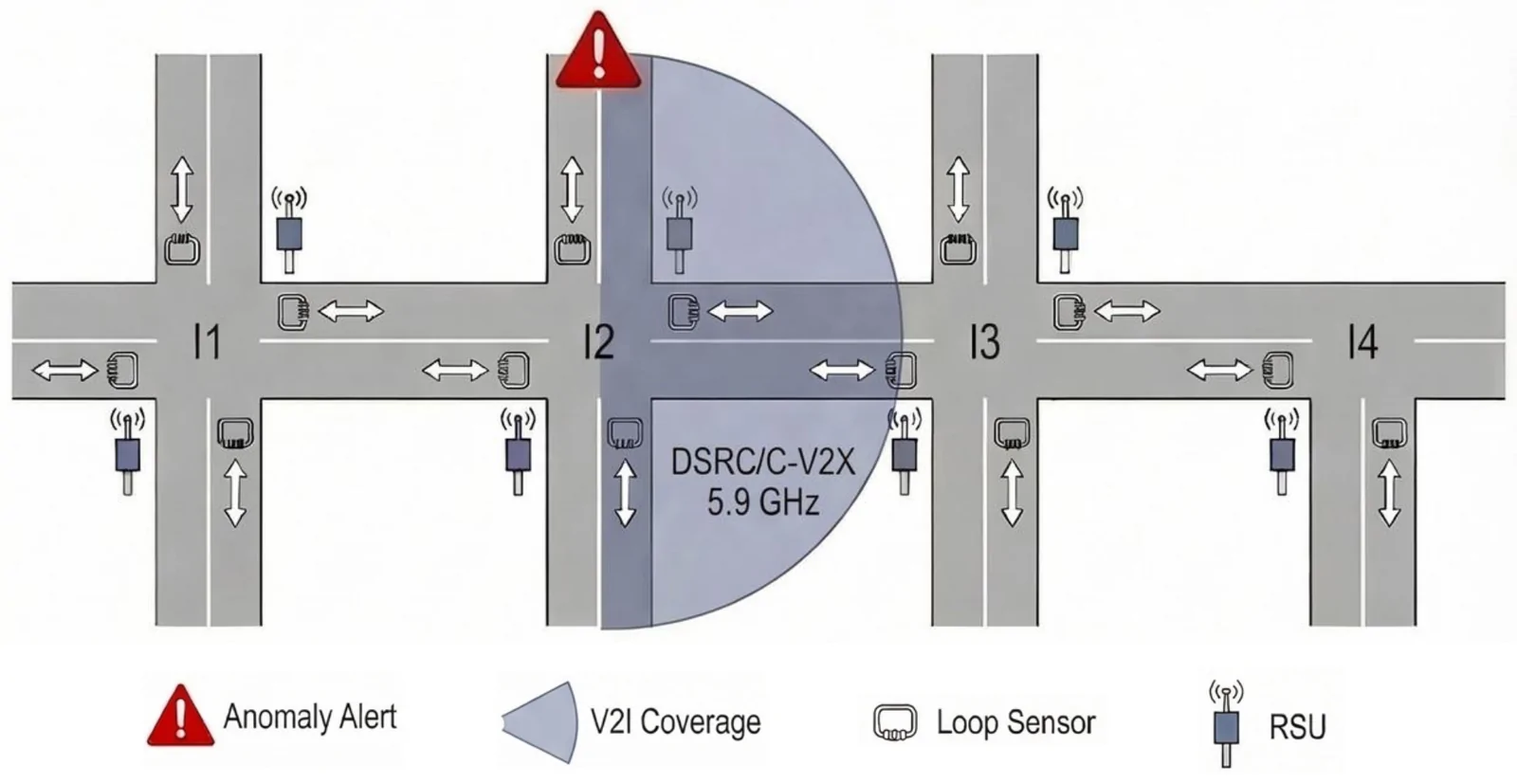
IoT-enabled V2I corridor across four consecutive signalized intersections (I1–I4). I4 is a three-way junction. Upon anomaly detection at I2, the co-located RSU broadcasts a safety alert via DSRC/C-V2X at 5.9 GHz within the shaded coverage arc.

Figure 1 illustrates the proposed corridor architecture. Each intersection hosts an inductive loop sensor embedded in the roadway and a co-located RSU mounted at the curbside. The directional flow arrows indicate the approach arms monitored at each site: four arms at I1–I3 and three at I4. The shaded arc at I2 represents the RSU broadcast coverage zone activated upon anomaly confirmation. The detection and alerting pipeline that governs data flow across these components is described in Section 3.3.

## 4. Methodology

This section presents the detection framework applied to the corridor dataset described in Section 3. The pipeline proceeds in four stages: data preprocessing, feature engineering, per-intersection anomaly detection via three benchmark methods, and corridor-level spatial reasoning.

### 4.1. Data Preprocessing

The raw sensor data arrived as hourly directional vehicle counts stored in wide format, with rows indexing hours and columns indexing days. Each intersection–direction pair was reshaped into long format with columns for intersection identifier, direction, date, hour, day type, and count. Day type was assigned according to the Gulf calendar, in which Friday and Saturday constitute the weekend and Sunday through Thursday are weekdays. Each directional stream was independently min–max normalized to the [0,1] interval. A frozen baseline was then computed per unique (stream, hour, day_type) triplet, yielding a mean μ and standard deviation σ derived exclusively from clean (non-anomalous) observations. That baseline was never refit on data containing injected or detected anomalies, thereby preventing data leakage. Concretely, calibration and evaluation were separated at the data level: baselines, feature scalers, and all detector training inputs were computed from the clean dataset before any injection occurs; the injected copies were used exclusively for evaluation; and no statistic derived from injected data fed back into baseline estimation, scaler fitting, or model training. Each baseline cell rested on a small sample by construction: two observations for weekend cells and three for weekday cells, with the sample standard deviation computed accordingly. Cells whose counts were identical across days yielded a zero standard deviation; in these flat cells, the z-score was defined as zero (the count necessarily equals the baseline mean there), which occurred in 3 of the 720 stream-hour-day-type cells in this dataset. No epsilon or variance floor was applied to small nonzero standard deviations, so small-sample noise in the baselines propagated into the z-scores and formed part of the five-day limitation discussed in Section 7. The northbound stream at I4 was excluded from directional-ratio features owing to the three-way intersection geometry, which produced 120 structural null values; all remaining features for I4 were computed normally across its three active approaches.

### 4.2. Feature Engineering

Fifteen engineered features were organized into three categories: temporal, directional, and spatial.

*Temporal features* were computed per stream per observation. A z-score quantified the deviation of each count from its frozen (hour, day_type) baseline. A percentage-of-baseline feature expressed the same deviation in relative terms. A three-hour rolling mean and rolling standard deviation captured short-horizon trend and volatility, respectively, while an hour-over-hour change delta encoded instantaneous acceleration. Hour-zero edge cases in the rolling window produced 20 null rows, which were excluded from rolling features only and did not affect point-wise features.

*Directional features* characterized the distribution of flow across approaches at each intersection–hour. Shannon entropy of the directional count vector peaked at 1.85–1.90 during busy hours, indicating approximately balanced four-way flow, and dropped at off-peak hours when a single approach dominated. An anomaly that blocked one approach arm would depress entropy during a typically high-entropy hour, providing a complementary detection signal. The directional imbalance ratio, defined as the maximum approach count divided by the total intersection count, captured the same asymmetry from the opposite perspective. North–south and east–west ratios were additionally computed for the four-way intersections but excluded for I4.

*Spatial features* operationalized the corridor-aware component of Contribution C2. Flow propagation ratios were defined between each adjacent pair: $r_{12}=V_{I2}/V_{I1}$, $r_{23}=V_{I3}/V_{I2}$, and $r_{34}=V_{I4}/V_{I3}$, where *V* denotes the total intersection volume at a given hour. Under normal conditions, adjacent intersections exhibited very high Pearson correlation ($\rho_{12}=0.984$, $\rho_{23}=0.974$, $\rho_{34}=0.907$), establishing a spatial baseline against which ratio deviations were flagged. A corridor standard deviation across the four intersection volumes at each hour provided a further indicator: elevated values signaled an uneven distribution inconsistent with the prevailing spatial profile. The spatial correlation structure aligns with the established finding that adjacent intersections in urban networks share highly correlated flow patterns [23]. The feature set combines static structural descriptors with dynamic temporal signals, following the joint spatiotemporal modeling paradigm advocated by Liu et al. [26].

### 4.3. Detection Methods

Three methods were benchmarked to span the spectrum from classical statistical monitoring to unsupervised machine learning and deep sequence modeling. Table 2 summarizes the rationale for each method’s inclusion and its expected operational strength.

**CUSUM.** The Cumulative Sum control chart was applied to the per-stream averaged z-score series in a two-sided formulation, maintaining separate upper and lower accumulators with slack parameter k=0.5 and decision threshold h=4.0 (both in units of the baseline standard deviation); both accumulators reset to zero after a flag event. The values follow standard statistical process control design guidance, which recommends a slack of half the shift magnitude deemed important and a decision interval of four to five standard deviations [40]. Appendix A reports a sensitivity grid over k∈{0.25,0.5,0.75,1.0} and h∈{3,4,5,6}, confirming that the adopted operating point lies in a flat performance region. CUSUM requires no training phase and incurs negligible computational cost, making it directly deployable on resource-constrained RSU edge hardware.

**Isolation Forest.** The Isolation Forest (200 estimators, fixed random state) was trained exclusively on the clean feature matrix, which comprised all 480 intersection-hour slots, including both weekday and weekend days, with rows containing undefined spatial-ratio features dropped; it was evaluated on the full feature matrix containing both normal and anomalous observations. The contamination hyperparameter was selected via grid search over {0.03,0.05,0.08,0.10,0.15}, with 0.03 yielding the best F1 score; Section 5.4 shows that the same value was independently re-selected when tuning was performed on injection seeds disjoint from the evaluation seeds. The lower contamination value is operationally appropriate for V2I deployments where each false alarm triggers an RSU broadcast, and the associated communication cost must be minimized. A key advantage of the Isolation Forest is that it requires no labeled anomalies, which is critical for real-world sensor deployments where ground-truth labels are unavailable. The method and its extended variant are well established in the anomaly detection literature [28,29,41], and recent IoT-specific benchmarks have confirmed their competitiveness on multivariate sensor data [30].

**LSTM Autoencoder.** The autoencoder comprised an encoder LSTM layer with 32 hidden units, a RepeatVector bridge of length 6, a decoder LSTM layer with 32 units, and a TimeDistributed Dense output layer. The network was trained with the Adam optimizer at its default learning rate of $10^{-3}$ and mean-squared-error loss, for at most 50 epochs with batch size 16, a 10% validation split, and early stopping on validation loss (patience 8, best weights restored). Inputs were four features (averaged z-score, total count, imbalance ratio, and directional entropy), min–max scaled with a scaler fitted on clean data only; NumPy and TensorFlow seeds were fixed at 42. Experiments used TensorFlow 2.21, NumPy 1.26.4, and scikit-learn 1.2.1. Because TensorFlow training is nondeterministic across identical runs unless operation determinism is enforced, repeated training runs of the same configuration varied on the order of ±0.005 F1. This training variability is distinct from the injection variability reported in Section 5.4: the multi-seed experiment evaluated a single trained model against ten injection sets, so the ±0.009 deviation in Table 5 reflects injection variability alone. The two sources are of comparable, small magnitude, and the multi-seed results remain the canonical LSTM-AE figures. Input sequences spanned six hours with a stride of one, yielding 460 training sequences from the clean partition, drawn from a five-day continuous hourly series per intersection with the sliding window applied across the full observation period without date-boundary resets, as hourly feature values were continuous across midnight transitions. The anomaly threshold was set at the 99th percentile of training reconstruction errors, selected via grid search across the 90th to 99th percentiles (maximum ΔF1 of 0.030 across this range); the same threshold was independently re-selected under the held-out tuning protocol of Section 5.4. With 460 training sequences, the reconstruction-error distributions for normal and anomalous windows exhibited limited separation, a characteristic that is quantified and discussed in Section 5. This architecture follows the LSTM autoencoder paradigm with statistical post-filtering [31] and is situated within the broader deep-learning framework for IoT time-series anomaly detection surveyed by Cook et al. [7] and Zamanzadeh Darban et al. [10].

### 4.4. Corridor-Level Spatial Reasoning

The corridor mechanism constitutes Contribution C2 and exploits the strong upstream–downstream correlation structure documented in Section 4.2. Detection proceeds in two stages. In the first stage, each method operates independently at the intersection–hour level, evaluating per-intersection z-scores and directional features to flag candidate anomalies. In the second stage, upon an anomaly flag at intersection $I_{n}$, the system queries the upstream intersection $I_{n-1}$. If $I_{n-1}$ is simultaneously flagged, the incident is inferred to originate upstream of $I_{n}$, and the RSU at $I_{n-1}$ broadcasts the alert. If $I_{n-1}$ is normal while $I_{n}$ is anomalous, the blockage is localized to the segment between $I_{n-1}$ and $I_{n}$, and the RSU at $I_{n}$ broadcasts. As reported in Section 5.6, this upstream query achieved correct localization for all detected corridor anomalies, conditional on the anomaly being flagged by the per-intersection detector. The upstream query mechanism therefore constitutes the operational implementation of Contribution C2, with the corridor-ratio features serving as supporting evidence for localization rather than as primary detection signals. The two-stage spatial reasoning aligns with graph-based approaches to road-network anomaly localization [24] and the integration of geographic corridor knowledge into diagnosis pipelines [42].

An ablation study examined whether directly incorporating raw corridor-ratio features into the Isolation Forest improved detection. As reported in Section 5.6, this approach degraded overall F1 and corridor-specific F1 relative to the z-score baseline, owing to extreme ratio values during single-intersection volume drops that pushed the IF decision boundaries incorrectly.

A dedicated CUSUM applied to ratio z-scores correctly activated the adjacent ratio channel but produced a high false-positive rate on normal slots driven by the natural variance of the I3-to-I4 ratio (standard deviation of 0.69 against a mean of 1.49).

An OR-fusion ensemble combining the ratio CUSUM with the z-score CUSUM similarly degraded performance. Full ablation results are reported in Table 7 and Section 5.6. These findings indicate that per-intersection ratio normalization or adaptive thresholding is required before ratio signals can serve as a standalone corridor detector; this refinement is identified as future work. The per-intersection z-score CUSUM already captures corridor anomalies effectively, rendering the raw-ratio ensemble redundant for the current dataset.

## 5. Results and Discussion

### 5.1. Dataset Characterization

**Figure 2:**
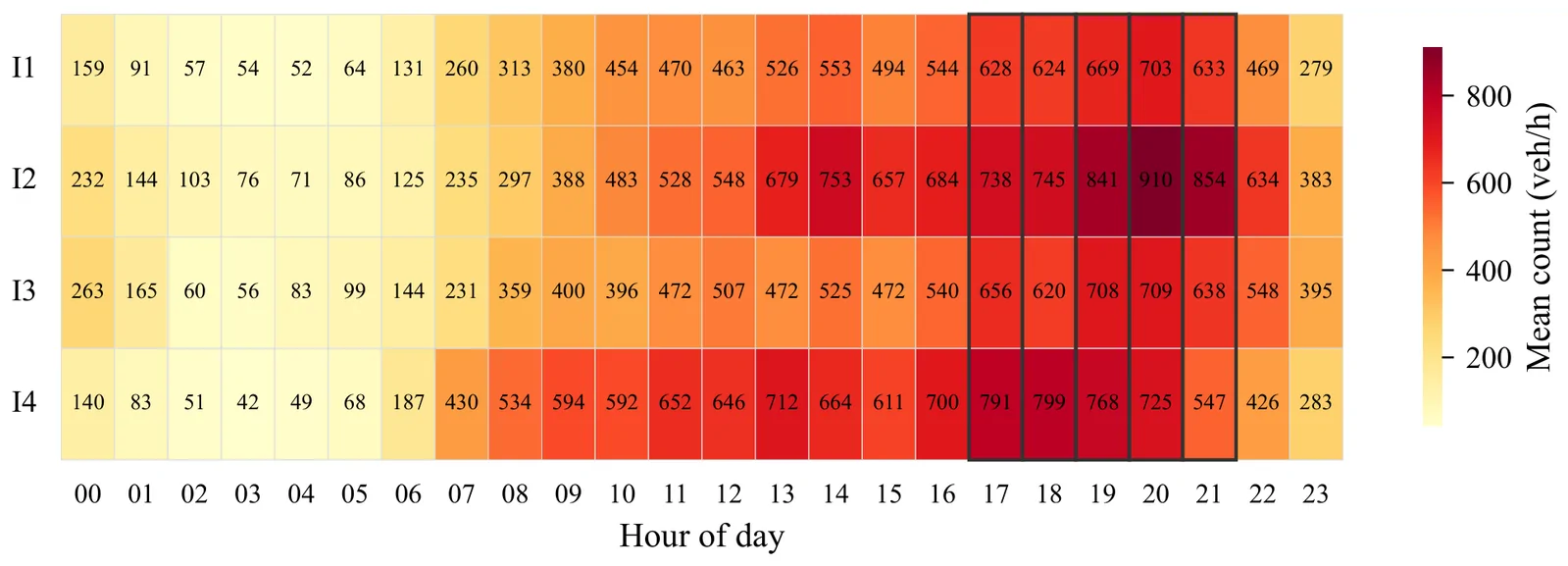
Mean hourly vehicle-count heatmap across I1–I4, averaged over the five-day observation period. Cell values indicate mean vehicles per hour.

The evaluation was conducted on the 1800-observation corridor dataset described in Section 3.2, spanning 15 directional streams over five days with a complete weekday–weekend regime split and no missing data. Under normal conditions, adjacent intersections exhibited high Pearson correlation of $\rho_{12}=0.984$, $\rho_{23}=0.974$, and $\rho_{34}=0.907$, as visualized in Figure 2. Peak-hour columns (17:00–21:00) show the evening-dominant profile characteristic of Gulf urban traffic, with I1–I3 peaking at 20:00 and I4 at 18:00. Notably, the I3↔I4 correlation degrades across the observation week from 0.956 on Friday to 0.840 on Tuesday, suggesting progressive weekday decoupling near the capacity-constrained three-way junction.

### 5.2. Anomaly Injection Methodology

The dataset contained no ground-truth anomaly labels, necessitating a synthetic injection protocol consistent with established evaluation practice for unlabeled real-world data [27,32]. Three anomaly types were injected into copies of the clean dataset. Point anomalies comprised 15 injections (eight drops and seven spikes): all directional counts at a randomly selected intersection–hour were multiplied by a factor drawn from U(0.15,0.35) for drops or U(2.5,3.5) for spikes, modeling sudden traffic incidents. Contextual anomalies consisted of 12 injections in which peak-hour slots were replaced with off-peak baseline values, testing the detector’s time-context awareness. Corridor anomalies comprised 10 injections in which all directional counts at a single intersection were multiplied by a factor drawn from U(0.20,0.40), corresponding to a 60–80% volume reduction, while adjacent intersections remained at normal levels, modeling an inter-intersection blockage. In total, 37 anomalies were injected across 480 intersection–hour slots, yielding an injection rate of 7.7%. The headline benchmark used random seed 42; Section 5.4 describes repeating the full protocol across ten independent seeds. Both weekend and weekday slots were eligible injection targets, and the clean data used for baselines and detector training included both day types, with separate frozen baselines maintained per (stream, hour, day type). Baselines were computed exclusively from clean data, ensuring no leakage between the injection and detection phases. As discussed in Section 7, these injected anomaly types are deviations relative to the frozen z-score baseline and are therefore well matched to a z-score change detector; this alignment should be borne in mind when interpreting the comparative ranking.

### 5.3. Detection Performance

**Figure 3:**
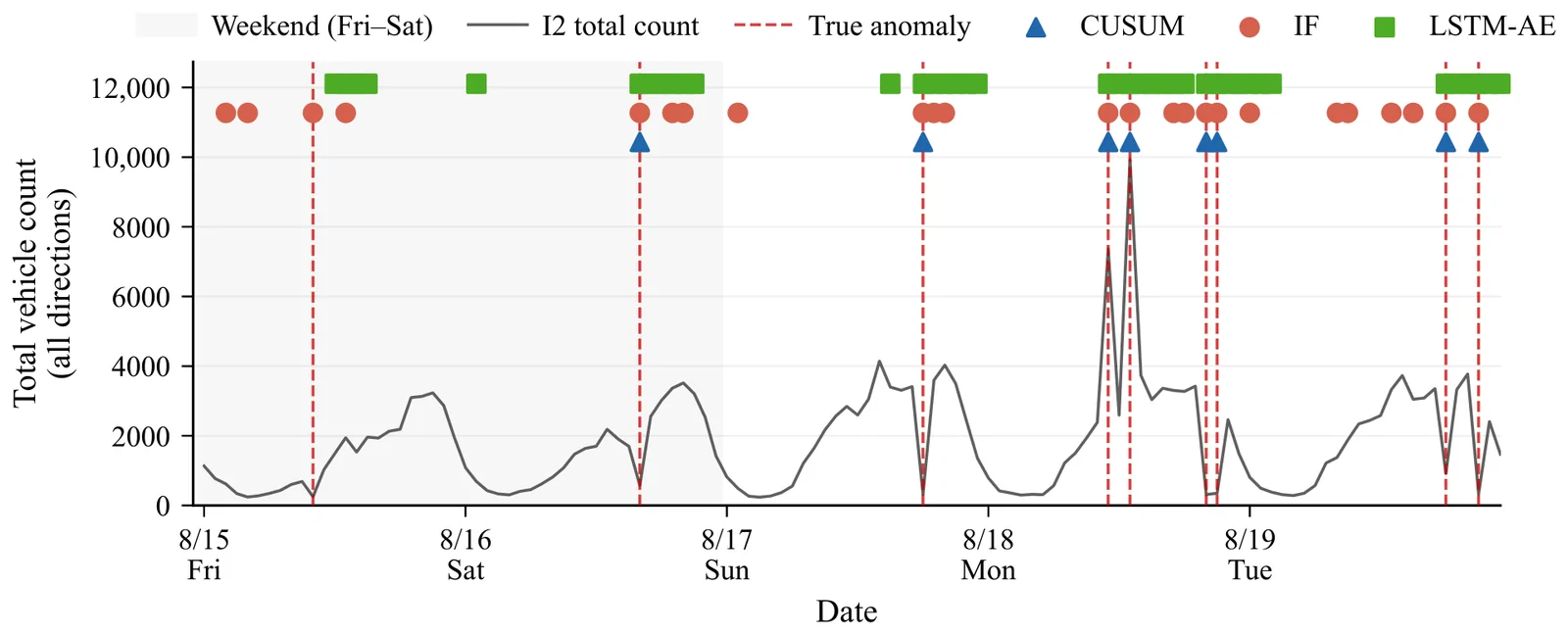
Detection timeline for intersection I2 (total vehicle count across all approach arms). Vertical dashed lines mark injected anomaly positions. Detection flags: CUSUM (Δ), Isolation Forest (○), LSTM-AE (☐). Shaded region indicates the Gulf-calendar weekend (Friday–Saturday).

Table 3 presents the overall detection performance of the three benchmark methods evaluated on the injected dataset. CUSUM achieves an F1 score of 0.958 with a precision of 1.000 and a recall of 0.919, outperforming both unsupervised alternatives, as illustrated in Figure 3. CUSUM flags align tightly with true anomaly positions, while Isolation Forest produces denser flags reflecting its higher false-positive rate at the default contamination parameter of 0.08. Perfect precision indicates that, on this injected set, every CUSUM alert corresponds to a genuine injected anomaly, a property of direct operational value in V2I broadcast deployments where each false alert incurs a communication cost and risks desensitizing downstream drivers.

**Figure 4:**
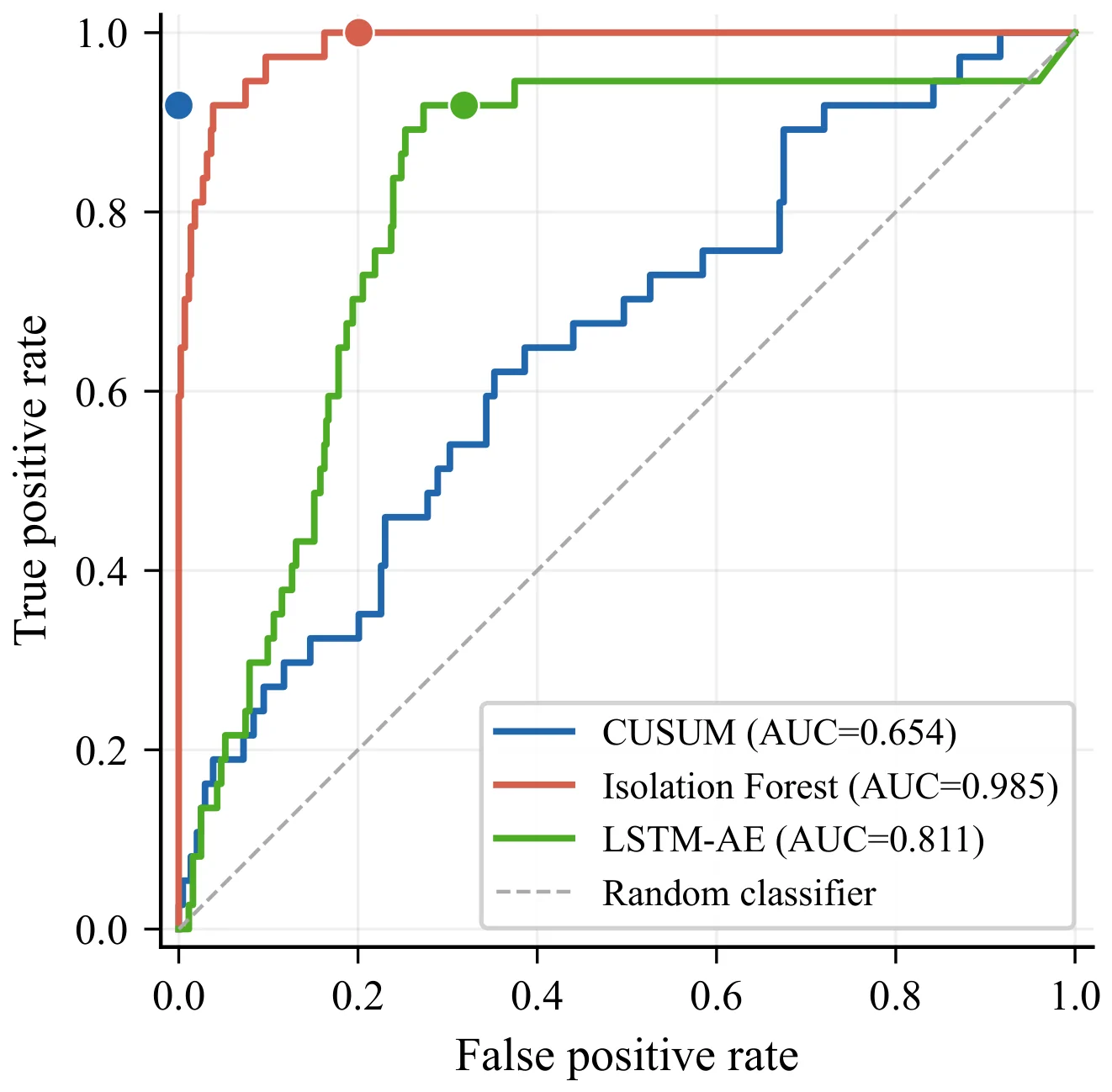
ROC curves for all three detection methods. Filled circles mark the operating points from Table 3.

At its default contamination parameter of 0.08, Isolation Forest yields an F1 of 0.454 with recall of 1.000 but precision of only 0.294, indicating systematic over-alerting. A grid search across contamination values {0.03,0.05,0.08,0.10,0.15}, with all other hyperparameters fixed (n_estimators = 200, random_state = 42), identifies 0.03 as optimal, improving F1 to 0.585 (ΔF1 =+0.131), precision to 0.419, and recall to 0.973. The monotonic improvement as contamination decreases confirms that the default setting over-estimates the true anomaly rate. At a contamination of 0.03, Isolation Forest misses only one of the 37 injected anomalies. Both configurations are reported in Table 3; the tuned setting supports the comparative claims, while the default is retained for transparency. The ROC curves for all three methods are presented in Figure 4, with operating points corresponding to Table 3. On a threshold-independent basis, Isolation Forest attains the highest area under the ROC curve (AUC = 0.985), ahead of the LSTM-AE (0.811) and CUSUM (0.654). This ordering inverts the operating-point ranking by F1 and precision, and the two measures are not in conflict. The area under the curve (AUC) rewards the ability to rank anomalies above normal observations across all thresholds, which favors the continuous scores of Isolation Forest and the reconstruction-error detector. CUSUM instead realizes its advantage at the calibrated decision threshold (h = 4.0), where it achieves perfect precision and the highest F1. Because the cumulative-sum statistic is a sequential change signal rather than a global ranking score, its swept-threshold ROC understates the operational performance attained at the chosen operating point.

**Figure 5:**
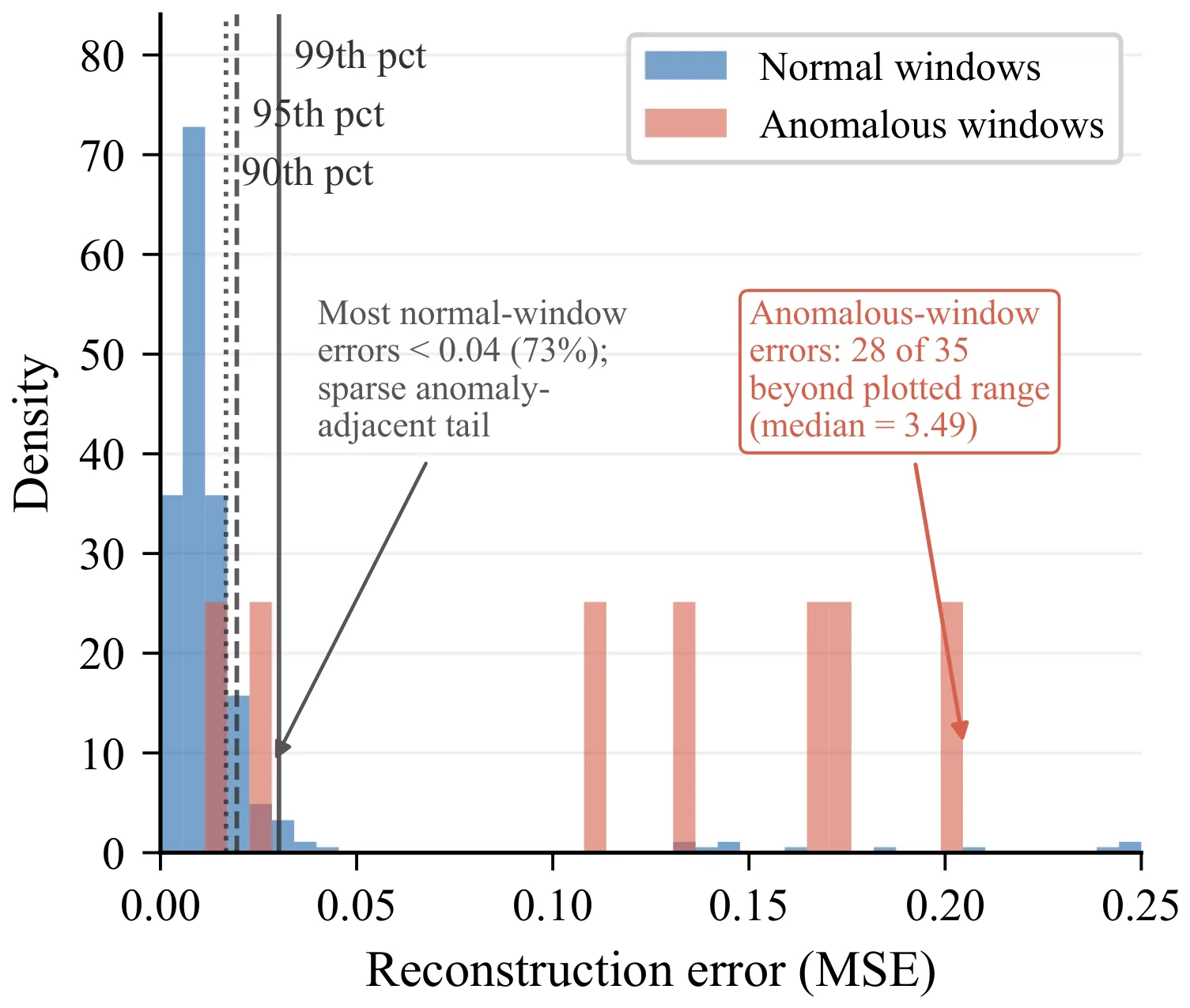
Reconstruction-error distributions for normal and anomalous windows under the LSTM-AE. Vertical dashed lines indicate the 90th, 95th, and 99th percentile thresholds evaluated during grid search.

The LSTM autoencoder exhibits limited sensitivity to threshold selection. A grid search across percentile thresholds from the 90th to the 99th yields a maximum ΔF1 of only 0.030, with the best configuration (99th percentile) achieving F1 = 0.351, precision = 0.219, and recall = 0.892. The reconstruction-error distributions of normal and anomalous windows are poorly separated, but not because anomalous errors are small: anomalous-window errors are large (median 3.49, with 28 of 35 beyond the plotted range of Figure 5), yet errors of comparable magnitude also occur in windows labeled normal, 27% of which exceed 0.04, driven by anomaly-adjacent windows within the sliding-window scheme. Large errors on both labels leave no clean decision boundary regardless of threshold placement. This behavior is consistent with a limited-training-data regime: with only five days of data (460 training sequences), the distributions do not separate cleanly. We refrain from asserting a specific minimum data volume, as it could not be verified with the available window; the LSTM-AE was retained in the benchmark to document this behavior and to inform deployments where longer collection windows are available.

Table 4 disaggregates CUSUM performance by anomaly type. CUSUM achieves F1 = 1.000 on both Point-Spike and Contextual categories. Corridor anomalies are detected at F1 = 0.947. Point-Drop detection reaches F1 = 0.857, with the two missed cases occurring exclusively during overnight floor-level hours (approximately 02:00–04:00), where baseline counts approach the sensor minimum of six vehicles per hour at the I4 westbound approach. A drop of 65–85% at these floor counts produces an absolute deviation too small for the CUSUM’s cumulative sum to cross the threshold h=4.0 within the observation window. This result delineates the method’s operating envelope: CUSUM is reliable during all waking and peak hours, with a quantified blind spot confined to overnight floor-level counts.

### 5.4. Robustness Across Injection Seeds and Held-Out Tuning

To establish that the comparative ranking was not an artifact of a single injection draw, the full protocol of Section 5.2 was repeated across ten independent injection seeds (42 through 51), regenerating all 37 anomalies per seed while holding the frozen baselines, feature scalers, and trained models fixed (all were functions of the clean data only and were therefore seed-independent). In addition to precision, recall, and F1, two threshold-independent metrics are reported: average precision (AP), the area under the precision–recall curve computed from each method’s continuous score (cumulative-sum magnitude, negated isolation score, and reconstruction error, respectively), and the Matthews correlation coefficient (MCC), which is robust to the 7.7% class imbalance of the injection protocol. Table 5 reports means and standard deviations. Because every seed re-injected anomalies into the same five-day traffic dataset, this experiment establishes robustness to the anomaly-injection process, that is, to anomaly placement and magnitude, not generalization to unseen traffic periods, which remains outside the reach of the present data (Section 7).

The ranking is stable: CUSUM leads on F1, AP, and MCC on every seed, with perfect precision throughout, and the seed-42 figures reported elsewhere in this paper lie within one standard deviation of the ten-seed means. The Isolation Forest and LSTM-AE recall figures confirm that both alternatives detect most injected anomalies but at substantially lower precision, consistent with the operating-point analysis of Table 3.

Hyperparameter selection was additionally repeated under a held-out protocol to rule out tuning-on-test optimism. Five tuning seeds (1001 through 1005), disjoint from the ten evaluation seeds, were used to regenerate independent injection sets; the Isolation Forest contamination grid and the LSTM-AE percentile grid of Section 4.3 were re-run on these tuning sets, and the configuration maximizing mean F1 across the tuning seeds was frozen before evaluation. The held-out procedure independently re-selected a contamination of 0.03 and the 99th percentile threshold, the same configuration used throughout this paper, and its performance on the evaluation seeds was identical to that in the tuned rows of Table 5. The reported figures are therefore not inflated by shared tuning and evaluation data.

### 5.5. Performance on Misaligned Anomaly Classes

The headline injection protocol perturbed counts as step changed relative to the frozen z-score baseline and was therefore structurally aligned with a z-score change detector (Section 7). To quantify how much of CUSUM’s advantage derived from that alignment, three additional anomaly classes were designed to depart from the step-change model and injected under the same frozen-baseline discipline, ten instances per class per seed across the ten evaluation seeds. *Gradual drift* ramped all directional counts at a random intersection linearly from unity to a factor drawn from U(0.5,0.7) over six hours, then held for three hours, with all nine affected hours labeled anomalous. *Sustained disruption with recovery* dropped counts to a factor from U(0.3,0.5) for three hours followed by a two-hour linear recovery, labeling all five hours. *Queue propagation* applied a drop drawn from U(0.2,0.4) at a downstream intersection and propagated attenuated echoes (attenuation 0.6 per hop) to the two upstream neighbors with one-hour staggers, modeling upstream queue formation. Detection delay was measured in hours from the first anomalous slot to the first flag within the anomalous window, over detected instances. Table 6 reports the results.

Two findings follow. First, the headline margin is indeed protocol-dependent: on gradual drift, CUSUM’s F1 falls to 0.640 with recall 0.472, and the LSTM-AE matches it (F1 0.644) with substantially higher recall (0.719) and a shorter mean detection delay (2.30 against 3.16 h). Where the anomaly signature departs from a step change, the deep detector closes the gap, which is consistent with the alignment limitation stated in Section 7 and indicates that the benchmark ranking should not be read as a general superiority claim. Second, CUSUM’s precision remains near unity on every class (0.985 to 0.997), so its distinguishing operational property, that alarms are almost never false, survives the change of anomaly model even when its recall does not. On sustained disruptions and queue propagation, whose onsets retain a step-like component, CUSUM remains the strongest overall (F1 0.734 and 0.792), while the LSTM-AE attains the highest recall on sustained disruptions (0.954). These results bound the conditions under which each method class is preferable and directly address the concern that the comparative design favors the change-point detector by construction.

### 5.6. Spatial Fusion Results

**Figure 6:**
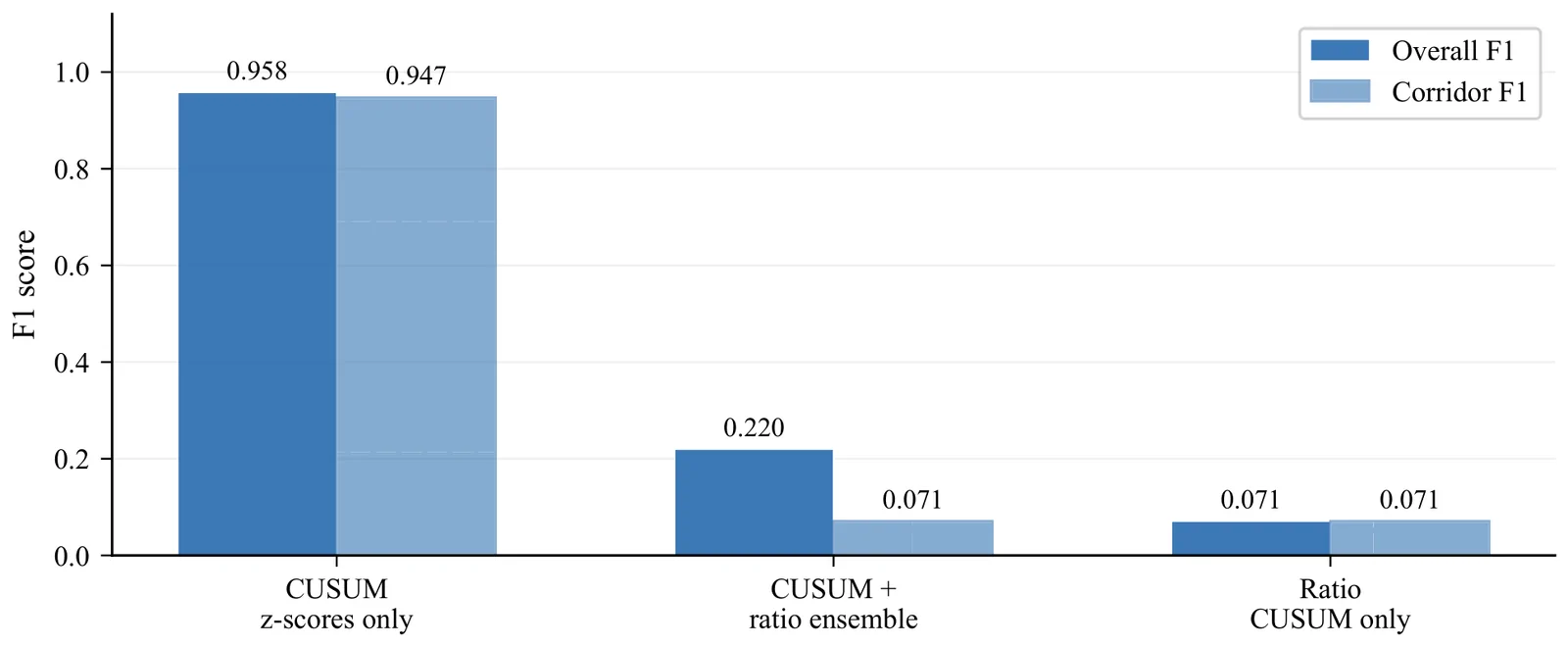
Spatial fusion ablation results. Solid bars represent overall F1; hatched bars represent corridor-type F1. See Table 7 for exact values.

Table 7 reports the effect of corridor-level spatial features. CUSUM applied to per-intersection z-scores alone achieves an overall F1 of 0.958 and a corridor-type F1 of 0.947. Adding raw corridor-ratio features via an OR-fusion ensemble degrades both metrics: overall F1 falls to 0.220 and corridor-type F1 to 0.071. A dedicated corridor-ratio CUSUM operating in isolation achieves an F1 of 0.071, with high natural variance in the I3-to-I4 ratio (standard deviation of 0.69 against a mean of 1.49) flagging 263 of the 443 normal evaluation slots as anomalous, corresponding to a false-positive rate of 59.4% (Figure 6). The sharp degradation from Config 1 to Config 2 confirms that raw ratio features destabilize the detector rather than improving corridor sensitivity.

The upstream query mechanism described in Section 4.4, by contrast, localizes detected corridor anomalies reliably. Localization is evaluated only on corridor anomalies that the per-intersection detector first flags, and a localization is counted as correct when the inter-intersection segment identified by the upstream-consistency rule matches the intersection at which the anomaly was injected. Of the 10 injected corridor anomalies, the per-intersection z-score CUSUM flagged nine (corridor-type F1 of 0.947 at unit precision; Table 4); the two-stage upstream query then localized all nine to the correct segment, that is, 100% localization accuracy conditional on detection. This figure therefore reflects a small, detection-conditioned sample and should not be read as evidence of reliable localization of real incidents. It confirms that the spatial correlation signal documented in Section 5.1 is operationally exploitable through the upstream query logic, but that the raw ratio features require per-intersection normalization or adaptive thresholding before they can serve as a standalone corridor detector.

### 5.7. Discussion

The experimental results support several deployment-oriented observations, subject to the limitations stated in Section 7.

Among the evaluated methods, CUSUM is the most accurate and the best suited to an edge-deployed detector, on the basis of three properties. Perfect precision on the injected set avoids wasted broadcasts (precision of 1.000 on every one of the ten injection seeds, F1 of 0.945±0.017; Section 5.4); negligible computational cost suits RSU edge hardware; and its F1 of 0.958 on the reference seed is the highest of any method evaluated. For high-sensitivity surveillance or logging deployments where missing an anomaly is more costly than investigating a false positive, the tuned Isolation Forest (contamination of 0.03) offers the highest recall of any method at 0.973, detecting 36 of 37 injected anomalies. The precision–recall tradeoff between CUSUM and tuned Isolation Forest reflects two distinct operational priorities that can coexist within the proposed two-tier architecture.

The computational suitability claim is quantified in Table 8. On a single thread, CUSUM processes one observation in under a microsecond with O(1) state per intersection; the Isolation Forest and LSTM-AE require roughly 54 and 75 microseconds per observation, respectively, plus a one-time training cost. All three are trivially feasible at the hourly cadence of Tier-2 even on modest hardware, so the hourly framework imposes no meaningful compute constraint. The distinction matters at Tier-1: only a detector with sub-microsecond per-event cost and constant memory, the profile of the streaming CUSUM, matches the per-vehicle-arrival cadence and is indicative of suitability for RSU-class edge hardware, which is why the Tier-1 design of Section 6.5 instantiates the CUSUM paradigm rather than the learned models; these figures were measured on workstation hardware, and benchmarking on deployment-class RSU devices is left to the field validation identified in Section 7.

The LSTM autoencoder’s underperformance is consistent with the available dataset size rather than an architectural limitation. Although the five-day window is too short to verify a specific data threshold, the poor reconstruction-error separation indicates that substantially longer collection windows would be required before the LSTM-AE’s capacity to capture temporal dependencies could be tested, subject to empirical validation on extended datasets. Consistent with this reading, the LSTM-AE matches CUSUM on the gradual-drift class (Section 5.5), where the anomaly signature departs from the step-change model; its disadvantage is specific to the headline protocol rather than uniform across anomaly types.

The evening-dominant traffic profile, peaking at 20:00 for I1–I3, and the 109% weekday volume lift at I4 reflect Gulf urban characteristics that are absent from the Western benchmark datasets predominant in the anomaly detection literature. The frozen z-score baselines capture this regional regime, with the tightest standard-deviation values occurring at weekday peak hours and the widest on weekend afternoons. This pattern supports the value of region-specific real-world data for ITS anomaly detection research and underscores the contribution of a Gulf-region physical traffic anomaly dataset to the field.

The five-day observation window captures a complete weekday cycle and a full weekend pattern, consistent with operational monitoring constraints in municipal IoT deployments. The framework is architecture-agnostic and designed to extend to longer observation periods. The injection-based evaluation methodology and frozen-baseline protocol, in which baselines are never refit on anomalous data, support the internal validity of the comparison within this observation period; broader generalization is left to future multi-corridor and longer-horizon studies, as discussed in Section 7. The comparative conclusions of this paper are accordingly claims about this corridor, this observation window, and this injection protocol; they are not presented as general statements about the relative merits of statistical and deep-learning detectors.

## 6. V2I Latency Feasibility Analysis

The hourly detection tier of Section 5 cannot meet the real-time requirements for safety-critical V2I alerting: the aggregation cadence alone exceeds the required lead time by multiple orders of magnitude. This section formalizes the latency constraint, quantifies the gap, derives a heuristic edge observation budget necessary for a complementary sub-second detection tier, and implements and evaluates that tier in simulation.

### 6.1. Latency Requirements

The binding latency constraint is derived from a worst-case approach scenario on the study corridor. At a conservative urban arterial speed of 80 km/h, a vehicle traverses the 300 m effective broadcast range of a standard DSRC roadside unit in 300÷(80,000÷3600)=13.5 s [12,15]. Any anomaly detection and alert broadcast sequence must therefore complete within 13.5 s to ensure that approaching vehicles receive the warning before entering the affected zone. A less demanding scenario, 40 km/h approach speed with 500 m extended range, yields a 45 s budget. The 13.5 s threshold is adopted throughout this analysis as the binding constraint because 80 km/h represents a conservative design speed for a high-density urban arterial of this classification [12]. DSRC and C-V2X transmission latencies in the 5.9 GHz ITS band are on the order of milliseconds and are therefore negligible relative to the detection latency [13,34].

The 13.5 s figure is a traversal-time budget, not a full latency model, and its composition warrants explicit statement. In deployment, the detection-to-warning chain comprises loop actuation and count aggregation at the signal controller, edge computation, message encoding and queuing at the RSU, over-the-air transmission, and in-vehicle processing. Field measurements place DSRC and C-V2X over-the-air and protocol-stack latencies at the millisecond scale under nominal load [13,14], and the Tier-1 detector’s per-arrival update is sub-microsecond (Table 8), so under nominal conditions, the budget is consumed almost entirely by detection, the quantity analyzed in this section. Under channel congestion at high vehicle densities, transmission latencies can grow to tens of milliseconds, and packet delivery degrades [14]. Because even a 50 ms communication allowance amounts to 0.4% of the 13.5 s budget, the conclusion that detection dominates is robust to realistic channel degradation. Alert delivery reliability under congestion, as distinct from latency, remains a deployment consideration outside the scope of this paper.

### 6.2. Detection Latency Gap

The relevant question at hourly granularity is not which detector is fastest but what the aggregation cadence itself permits. When an hourly detector catches an injected anomaly, it does so in the anomaly’s own slot (Table 3), but a same-slot flag is not a fast alert: the hourly count for a slot does not exist until the hour completes, so the wall-clock delay from anomaly onset to the earliest possible alert is set by the aggregation cadence. For an onset uniformly distributed within the hour, the expected delay is approximately 1800 s, with a worst case of 3600 s for an onset at the start of an hour. As Table 9 reports, this structural floor exceeds the 13.5 s alerting budget by a factor of roughly 133 in expectation and 267 in the worst case, independent of which detection algorithm runs on the aggregated counts. The gap is therefore a structural consequence of hourly temporal aggregation, not a deficiency of the detection algorithms themselves: the methods are designed to operate on hourly IoT sensor data and fulfill a different role in the proposed architecture. This finding motivates the two-tier design introduced below.

### 6.3. Two-Tier Architecture

The latency gap establishes that no hourly detection method can serve as a standalone V2I safety alerting mechanism. The proposed engineering response is a two-tier architecture in which each tier addresses a distinct temporal scale.

Tier-1 is a sub-second streaming detector deployed at the RSU edge, designed to meet the 13.5 s latency budget by operating directly on individual vehicle arrivals. Section 6.5 implements this tier and evaluates it in simulation calibrated to the observed arrival rates.

Tier-2 is the hourly IoT detection framework described in Section 4 and Section 5. Its operational role within the two-tier architecture is to provide calibration inputs to the Tier-1 edge detector. These inputs comprise three concrete data products: (a) learned normal baseline profiles, that is, per-intersection hourly mean and standard-deviation parameters derived from the frozen-baseline protocol described in Section 4; (b) the validated anomaly-class definitions and magnitude distributions, together with the precision-first operating philosophy expressed as a false-alarm budget rather than as fixed thresholds; and (c) spatial correlation parameters ($\rho_{12}=0.984$, $\rho_{23}=0.974$, $\rho_{34}=0.907$) for upstream-consistency checking as characterized in Section 5.6. This calibration relationship, in which the hourly framework continuously refines the inputs of the sub-second edge layer, defines the operational role of the Tier-2 framework within the proposed two-tier architecture. To be explicit about the division: Tier-2 supplies the arrival-rate baselines, the regime structure, the anomaly-class definitions, and the false-alarm target, whereas the Tier-1 decision thresholds themselves are calibrated separately at the edge for the continuous-time detector (Section 6.5), because thresholds tuned for hourly z-score accumulation do not transfer to a per-arrival exponential CUSUM.

### 6.4. Edge Observation Budget

A heuristic observation-budget design target for a Tier-1 edge detector was derived from the measured traffic arrival rates. The mean peak arrival rate across the four intersections during peak hours (18:00–20:00) was 0.764 vehicles per second, computed by multiplying per-intersection hourly counts by the number of active approach arms (four for I1–I3, three for I4), dividing by 3600, and averaging across intersections (I1 = 0.739, I2 = 0.924, I3 = 0.754, I4 = 0.637 veh/s). A minimum of nine vehicle observations was adopted as a heuristic design target for the Tier-1 detector, motivated by statistical process control practice in which subgroup sizes on the order of four to ten observations support control-chart comparisons at conventional three-sigma limits [40]. This figure is a design heuristic rather than a derived sample-size requirement; its adequacy is assessed empirically in Section 6.5, whose measured detection latencies supersede the analytic budget as the operative feasibility evidence. At the mean arrival rate, accumulating nine vehicles required 9÷0.764=11.8 s. The resulting design target for the Tier-1 detector was a window of approximately 11.8 s or nine vehicles, whichever condition was satisfied first.

The per-intersection analysis revealed an asymmetry. I2, with the highest arrival rate of 0.924 veh/s, reached the nine-vehicle threshold in approximately 9.7 s, comfortably within the 13.5 s latency budget. I4, however, had the lowest arrival rate at 0.637 veh/s and required approximately 14.1 s to accumulate nine vehicles, marginally exceeding the budget. The three-way geometry and lower traffic volume at I4 make it the binding constraint for Tier-1 deployment. This finding carries direct practical implications: RSU configuration at I4 may require either a reduced statistical threshold or an extended broadcast range to compensate for the slower vehicle accumulation rate. To the authors’ knowledge, an edge observation budget of this kind had not previously been quantified for physical traffic anomaly detection in a V2I corridor context.

### 6.5. Tier-1 Detector Evaluation in Simulation

To assess whether the observation budget derived above was achievable in operation, the Tier-1 detector was implemented and evaluated in simulation calibrated to the observed corridor. Per-vehicle arrivals at each intersection were modeled as piecewise-homogeneous Poisson streams whose baseline rates were computed from the recorded hourly totals across approach arms in the clean dataset. This arrival model abstracts away signal-cycle platooning, so the Poisson results should be read as a simulation-level bound; a platooned-arrival sensitivity analysis is reported at the end of this subsection. Evaluation was stratified into three weekday rate regimes per intersection, defined as the mean rate over the three highest (peak), three middle (off-peak), and three lowest (floor) hours of the weekday profile. The resulting peak-regime rates ranged from 0.80 to 1.04 veh/s, off-peak rates from 0.58 to 0.69 veh/s, and floor rates from 0.040 to 0.084 veh/s. These regime rates are weekday-only and specific to each intersection’s own extreme hours; they therefore exceed the five-day 18:00–20:00 corridor means used in the observation-budget derivation of Section 6.4, and the two quantities are reported separately.

The Tier-1 detector consisted of two one-sided continuous-time exponential CUSUM charts per intersection. The rate-decrease chart tested against an alternative rate of one quarter of the baseline rate and accumulated evidence between arrivals, so that lengthening inter-arrival gaps drove the statistic upward; threshold crossings between arrivals were computed exactly in an event-driven manner. The rate-increase chart tested against an alternative of three times the baseline rate and accumulated evidence at arrival instants. Detection thresholds were calibrated separately for each intersection and regime by searching on clean simulated traffic to a false-alarm target of at most 0.1 alarms per hour per chart, consistent with the precision-first operating point adopted for the hourly tier; calibrated thresholds ranged from 6.0 to 10.0.

Sub-second anomalies were injected as rate multipliers mirroring the hourly injection protocol: flow cutoff with multiplier U(0.15,0.35), surge with U(2.5,3.5), and partial degradation with U(0.50,0.70). Each trial applied the anomalous rate for 120 s from a random onset following a 30-min warmup during which the chart state evolved under clean traffic. Detection latency was measured from onset to alarm, undetected trials were scored as exceeding the alerting budget, and 200 replications were run per intersection, regime, and anomaly class with a fixed random seed.

**Figure 7:**
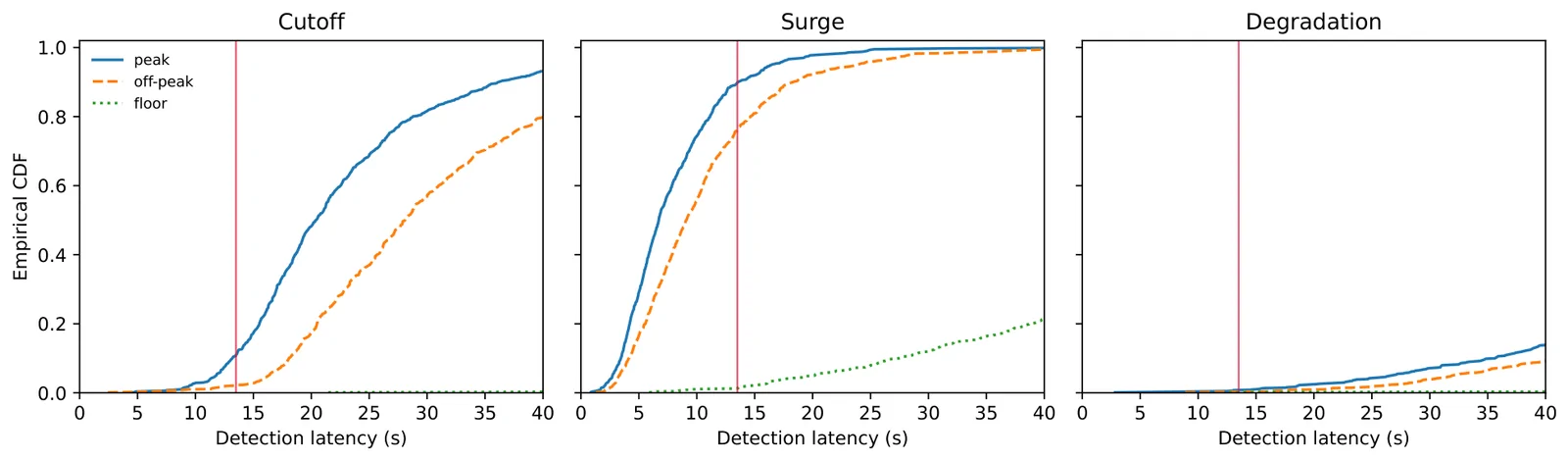
Empirical detection-latency CDFs of the Tier-1 detector by rate regime for each anomaly class, pooled across intersections and computed over all trials, so each curve saturates at its class detection rate. The vertical line marks the 13.5 s V2I alerting budget at 80 km/h.

Table 10 and Figure 7 summarize the results. Surge-type anomalies were detected in every trial at operational rates and met the 13.5 s alerting budget in 89.8% of peak-regime trials (pooled median latency 6.8 s, 90th percentile 13.3 s) and 75.9% of off-peak trials (median 9.1 s). Flow cutoffs were also detected in essentially every trial at operational rates, but at a pooled median latency of 20.5 s in the peak regime and 27.9 s off peak, approximately 1.5 to two times the alerting budget, with only 2% to 11% of trials within budget. Partial degradation was detected in at most 50.8% of trials at operational rates, with median latencies near one minute. In the floor regime, all three classes fell far outside the budget, and the lowest arrival rates produced the most extreme individual failures: at intersection I4, whose floor-regime rate was 0.040 veh/s, the detection rate was 0.035 for cutoffs and 0.005 for degradation. This extends the overnight blind-spot finding of the hourly analysis to sub-second granularity. Across intersections, the mean within-budget rates were closely clustered, so I4 sat in the bottom cluster but was not uniformly the worst case.

The asymmetry between surge and cutoff detection is fundamental rather than a tuning artifact. Evidence for a rate increase occurs with every additional vehicle at the elevated rate, whereas evidence for a rate decrease accrues only through the absence of expected arrivals and is therefore bounded by the baseline rate. With threshold *h* and baseline rate $\lambda_{0}$, even a total cessation of flow cannot cross the threshold in less than approximately $h/\lambda_{0}$ s, which is roughly 9 to 12 s at the observed peak-regime rates. The observed cutoff medians of roughly 20 s for partial cutoffs are consistent with this bound. Relaxing the false-alarm target would shorten latencies for all classes at the cost of alarm precision; the strict target was retained to match the operating philosophy established for the hourly tier, where every broadcast alert carries a communication and driver-attention cost.

To probe the Poisson assumption directly, the full experiment was repeated under a platooned arrival process: a fixed 90 s signal cycle with an effective green fraction of 0.45, arrivals occurring only during green phases as Poisson with rate $\lambda_{0}/0.45$ so that the mean rate was preserved, and detection thresholds recalibrated under the platooned process to the same 0.1 alarms-per-hour target. Platooning raised the calibrated rate-decrease thresholds sharply, with several exceeding 30, because clean-traffic red phases produced long arrival gaps that drove the rate-decrease chart upward and calibration had to absorb this cyclic drift. The net effect on detection was directionally favorable or neutral rather than adverse: surge within-budget rates changed by at most a few percentage points, while cutoff within-budget rates improved by roughly 31 percentage points at operational regimes, because a genuine cutoff began from a chart state already elevated by the preceding red phase. Floor-rate hours remained the binding blind spot under both arrival processes. Two implications follow. First, the headline Poisson results are conservative rather than optimistic for cutoff detection and essentially unchanged for surges, so the qualitative conclusions of this subsection are robust to signal-cycle arrival structure. Second, the magnitude of the recalibrated thresholds indicates that a detector conditioning explicitly on signal phase, rather than absorbing the cycle into a higher threshold, is the appropriate next design step; the present analysis establishes robustness to platooning, not a validated cycle-aware design.

Relative to the hourly tier’s structural floor, the Tier-1 detector reduced detection latency by a factor of roughly 265 for the surge class (6.8 s against the 1800 s expected hourly delay in Table 9) and roughly 90 for cutoffs. The simulation-level conclusion is therefore that the two-tier architecture closes the latency gap for surge-type anomalies at operational traffic rates, while rate-decrease anomalies remain detection-limited under strict false-alarm control, and low-rate overnight hours remain a structural blind spot at any granularity. Corroboration across adjacent intersections, which the corridor correlation structure of Section 5.1 supports, is a natural direction for reducing cutoff latency without relaxing per-intersection false-alarm targets.

## 7. Limitations and Threats to Validity

Several limitations bound the scope and interpretation of these findings. First, the dataset spanned five consecutive days at a single four-intersection corridor; it captured one complete Gulf weekday–weekend cycle but not weekly or seasonal variability, special events, or longer-term demand shifts, so the conclusions are best read as a preliminary, corridor-specific case study rather than as evidence of deployment readiness. Second, the traffic counts were real but the anomaly labels were not: verified incident records were unavailable, so all reported scores measure the detectability of injected anomalies rather than performance on real incidents. Third, the injected step-change anomalies were structurally well matched to a z-score change detector; Section 5.5 quantified this alignment effect, showing that the margin narrowed substantially on gradual drift, and that the headline ranking was therefore protocol-dependent.

Fourth, the corridor-aware component improved localization rather than detection: raw corridor-ratio features reduced overall F1 from 0.958 to 0.220 (Section 5.6), so the spatial mechanism functions as a post-detection localization rule and not as a spatial-fusion detector. Fifth, the reported 100% localization accuracy is conditional on prior detection and rests on only nine detected corridor anomalies of ten injected; it should not be generalized to real-incident localization. Sixth, the Tier-1 detector was evaluated in simulation rather than on field data, with synthetic sub-second anomalies and Poisson arrivals that abstracted away signal-cycle platooning, although the platooned-arrival sensitivity analysis of Section 6.5 indicated that these results were conservative for cutoff detection and stable for surges; they established simulation-level feasibility, not an operational demonstration. Finally, the LSTM-AE data-requirement discussion is an interpretation of the observed reconstruction-error overlap and was not verified experimentally with the available data. Addressing these limitations, chiefly through a larger multi-corridor dataset, independently verified anomaly labels, and field validation of the Tier-1 detector under real arrival processes, defines the principal directions for future work.

## 8. Conclusions

This paper examined multi-intersection physical traffic anomaly detection on IoT loop-sensor data with an integrated V2I alert latency analysis, evaluated on real-world data from a high-motorization Gulf city. A five-day dataset of 1800 hourly directional vehicle-count observations from four consecutive signalized intersections in Kuwait City served as the evaluation testbed. The first, second, and fourth findings were demonstrated on real count data under synthetic injection, whereas the Tier-1 component of the third was demonstrated in simulation calibrated to that data.

First, the paper provided, to our knowledge, the first comparative benchmark of CUSUM, Isolation Forest, and LSTM autoencoder on Gulf-region urban IoT vehicle-count data (C1). CUSUM achieved an F1 score of 0.958 with perfect precision on the injected set (0.945±0.017 across ten injection seeds, precision of 1.000 on every seed), making it the most accurate method evaluated and the best suited to edge deployment. Tuned Isolation Forest (contamination of 0.03) attained the highest recall at 0.973, missing only one of 37 injected anomalies, and is therefore suited to high-sensitivity surveillance contexts. The LSTM autoencoder achieved an F1 of 0.351, with underperformance consistent with the limited training data available rather than the architecture itself, as characterized by the reconstruction-error distribution analysis. On deliberately misaligned anomaly classes the margin narrowed, and the LSTM-AE matched CUSUM on gradual drift, confirming that the headline ranking is specific to the injection protocol.

Second, a corridor-aware upstream query mechanism (C2) exploited inter-intersection spatial correlations (Pearson *r* from 0.907 to 0.984 across adjacent pairs) to localize detected anomalies to specific inter-intersection segments, achieving 100% localization accuracy on the nine of ten injected corridor anomalies that were first detected. An ablation showed that raw corridor-ratio features degraded detection, so the spatial component functions as a post-detection localization step.

Third, a V2I latency feasibility analysis (C3) quantified the detection-to-broadcast gap against the 13.5 s alerting requirement at 80 km/h. Hourly aggregation alone imposed an expected detection delay of approximately 1800 s, exceeding that constraint by two orders of magnitude regardless of detector and motivating a two-tier architecture. The analysis derived a heuristic Tier-1 observation-budget design target of 11.8 s (nine vehicles), with intersection I4 identified as the binding constraint at 14.1 s owing to its three-way geometry and lower arrival rate of 0.637 veh/s. A sub-second Tier-1 CUSUM detector was then implemented and evaluated in simulation calibrated to the observed arrival rates. Surge-type anomalies were detected well within the 13.5 s budget (pooled median 6.8 s at peak rates), flow cutoffs were detected reliably but at roughly 1.5 times the budget under a false-alarm target of 0.1 alarms per hour, and low-rate overnight hours remained a structural blind spot, extending the hourly blind-spot finding to sub-second granularity.

Fourth, this study constitutes, to our knowledge, the first comparative physical traffic anomaly-detection benchmark on real-world IoT vehicle-count data from a high-motorization Gulf city (C4), revealing an evening-dominant traffic profile (peak at 20:00 for I1–I3) that contrasts with patterns typical of Western benchmark datasets.

As detailed in Section 7, the principal limitations are the five-day single-corridor window, the use of synthetically injected rather than independently verified anomaly labels, and a Tier-1 detector evaluated in simulation rather than on field data. Future work will therefore pursue field validation of the Tier-1 detector under real arrival processes, corridor-pooled detection to reduce cutoff latency without relaxing per-intersection false-alarm targets, evaluation against independently verified anomaly labels, and multi-corridor deployment to assess transferability across varying intersection geometries and traffic regimes, where privacy-preserving mechanisms for vehicular sensor data sharing will become an additional design consideration [43]. Five days at one corridor were sufficient for this paper’s stated purpose, a proof-of-concept benchmark of the two-tier framework on one complete Gulf weekday–weekend cycle; they are not presented as sufficient for generalization, which requires the multi-corridor, longer-horizon validation identified above. Overall, within the scope of this corridor, observation window, and injection protocol, the results indicate that classical statistical methods remain competitive for physical anomaly detection on real-world IoT sensor data. Corridor-aware localization combined with a V2I latency analysis further provides a calibration and monitoring framework that can inform a future V2I safety-alerting system in high-density urban environments where ground-truth anomaly data are unavailable.

## Figures and Tables

**Table 1:** Comparison of related work on physical traffic anomaly detection.

Study	Sensor	Scope	Multi-Int.	V2I Lat.	Region
Rizvi [8]	Loop	Single	No	No	Western
He [2]	Count	Single	No	No	Western
Mao [1]	GPS/camera	Network	No	No	W./Asian
Nouri [19]	Count	Network	No	No	Western
Alzamzami [11]	Camera	Single	No	No	Gulf
**This work**	**IoT loop**	**Corridor**	**Yes**	**Yes**	**Gulf**

**Table 2:** Summary of benchmark detection methods.

Method	Mechanism	Inclusion Rationale	Deployment Scenario
M1: CUSUM	Sequential change-point detection on z-scores; flag when cumulative deviation exceeds threshold	Fast, interpretable, no training required; realistic for RSU deployment	Best detection latency for real-time use
M2: Isolation Forest	Unsupervised ensemble isolating anomalies via random recursive partitioning	No labeled anomalies in real data; handles multivariate features natively	High-sensitivity surveillance; minimizes missed detections
M3: LSTM-AE	Autoencoder trained on normal sequences; anomaly score equals reconstruction error	Captures temporal dependencies; strong baseline for deep-learning comparison	Detects subtle pattern deviations given sufficient data

**Table 3:** Overall detection performance. Both Isolation Forest configurations are listed; the tuned setting (contamination of 0.03) supports the comparative claims in this paper, while the default setting (0.08) is retained for transparency. Detection, when it occurs, is concurrent with the anomalous slot at hourly granularity; wall-clock alerting latency is governed by the aggregation cadence and is analyzed in Section 6.

Method	Precision	Recall	F1
CUSUM	1.000	0.919	0.958
Isolation Forest (default, c=0.08)	0.294	1.000	0.454
Isolation Forest (tuned, c=0.03)	0.419	0.973	0.585
LSTM-AE (99th percentile)	0.219	0.892	0.351

**Table 4:** F1 score by anomaly type.

Method	Point-Drop	Point-Spike	Contextual	Corridor
CUSUM	0.857	1.000	1.000	0.947
Isolation Forest	0.152	0.136	0.212	0.183
LSTM-AE	0.090	0.078	0.145	0.112

**Table 5:** Detection performance across ten injection seeds (mean ± standard deviation). CUSUM attains a precision of 1.000 on every individual seed.

Method	Precision	Recall	F1	AP	MCC
CUSUM	1.000 ± 0.000	0.896 ± 0.031	0.945 ± 0.017	0.987 ± 0.006	0.943 ± 0.018
Isolation Forest	0.420 ± 0.026	0.955 ± 0.025	0.583 ± 0.024	0.813 ± 0.060	0.592 ± 0.022
LSTM-AE	0.213 ± 0.008	0.944 ± 0.034	0.347 ± 0.009	0.270 ± 0.039	0.365 ± 0.009

**Table 6:** Performance on deliberately misaligned anomaly classes (means over ten injection seeds). Delay is the mean time from anomaly onset to first flag, over detected instances.

Class	Method	F1	Prec.	Rec.	AP	MCC	Delay (h)
Gradual drift	CUSUM	0.640	0.997	0.472	0.833	0.647	3.16
	Isolation Forest	0.516	0.661	0.427	0.625	0.449	3.57
	LSTM-AE	0.644	0.585	0.719	0.539	0.557	2.30
Sustained + recovery	CUSUM	0.734	0.997	0.582	0.827	0.743	0.30
	Isolation Forest	0.568	0.532	0.614	0.599	0.517	0.24
	LSTM-AE	0.705	0.560	0.954	0.631	0.693	0.18
Queue propagation	CUSUM	0.792	0.985	0.663	0.913	0.788	0.21
	Isolation Forest	0.616	0.554	0.697	0.706	0.559	0.04
	LSTM-AE	0.455	0.311	0.845	0.313	0.404	0.07

**Table 7:** Spatial fusion ablation results. Upstream-query localization: 9/9 correct on detected corridor anomalies (9 of 10 injected detected overall); see text.

Configuration	Precision	Recall	F1	Corridor F1
CUSUM z-scores only	1.000	0.919	0.958	0.947
CUSUM + ratio ensemble	0.123	1.000	0.220	0.071
Ratio CUSUM only	0.037	1.000	0.071	0.071

**Table 8:** Computational cost (single-threaded; inference over the 480-slot evaluation pass, median of 20 repeats). LSTM-AE setup time is training to early stopping.

Method	Setup (s)	Inference per Pass (ms)	Per Observation (μs)
CUSUM	0.000	0.42	0.87
Isolation Forest	0.11	25.8	53.8
LSTM-AE	4.75	35.9	74.9

**Table 9:** V2I detection latency gap imposed by hourly aggregation. The delay floor is detector-independent: any method operating on hourly counts inherits it.

Hourly Aggregation Delay	Delay (s)	Required Lead Time (s)	Gap
Expected (onset uniform within hour)	1800	13.5	133×
Worst case (onset at start of hour)	3600	13.5	267×

Required lead time computed at 80 km/h and 300 m RSU range.

**Table 10:** Tier-1 simulation results pooled across I1–I4 (200 replications per intersection, regime, and class; false-alarm target 0.1 per hour per chart).

Class	Regime	Det.	≤13.5 s	Med. (s)	p90 (s)
Cutoff	Floor	0.155	0.000	102.2	114.2
Cutoff	Off-peak	0.999	0.021	27.9	49.0
Cutoff	Peak	1.000	0.110	20.5	36.1
Surge	Floor	0.804	0.013	59.4	102.1
Surge	Off-peak	1.000	0.759	9.1	18.4
Surge	Peak	1.000	0.898	6.8	13.3
Degradation	Floor	0.026	0.001	84.3	99.2
Degradation	Off-peak	0.393	0.003	64.5	102.3
Degradation	Peak	0.508	0.008	59.4	106.6

Median and p90 latencies were computed over detected trials; trials undetected within the 120 s window were scored as exceeding the budget.

## Data Availability

The vehicle-count data analyzed in this study are subject to access restrictions and are not publicly available; they may be obtained from the author upon reasonable request and with the permission of the relevant traffic authority. The synthetic anomaly-injection procedure and analysis code are available from the author upon reasonable request.

## Appendix A. Sensitivity Analyses

Table A1 reports mean F1 across the ten evaluation seeds for a grid over the CUSUM slack *k* and decision threshold *h*. Performance is flat in the neighborhood of the adopted operating point (k=0.5, h=4.0; mean F1 0.945±0.017): the best grid cell (k=0.5, h=3.0; mean F1 0.963±0.017) lies within one standard deviation of it. The adopted values, chosen a priori from statistical process control convention [40], are retained throughout the paper to avoid post hoc selection on the evaluation seeds.

**Table A1:** CUSUM sensitivity grid: mean F1 over ten injection seeds.

	h=3	h=4	h=5	h=6
k=0.25	0.891	0.909	0.896	0.890
k=0.5	0.963	0.945	0.916	0.897
k=0.75	0.955	0.933	0.910	0.891
k=1.0	0.951	0.928	0.900	0.885

Table A2 reports the CUSUM detection rate as a function of injected point-anomaly magnitude (default k=0.5, h=4.0; ten injections per magnitude across five seeds). Detection is near-saturated for severe drops (multiplier at or below 0.2) and strong spikes (multiplier at or above 2.5), degrades smoothly through moderate magnitudes, and collapses for mild drops (multiplier at or above 0.8), quantifying the detector’s operating envelope with respect to anomaly severity.

**Table A2:** CUSUM detection rate versus point-anomaly magnitude multiplier.

Drop multiplier	0.1	0.2	0.3	0.4	0.5	0.6	0.7	0.8	0.9
Detection rate	0.92	0.92	0.80	0.78	0.70	0.64	0.60	0.30	0.06
Spike multiplier	1.5	2.0	2.5	3.0	3.5				
Detection rate	0.78	0.94	0.98	1.00	1.00				
